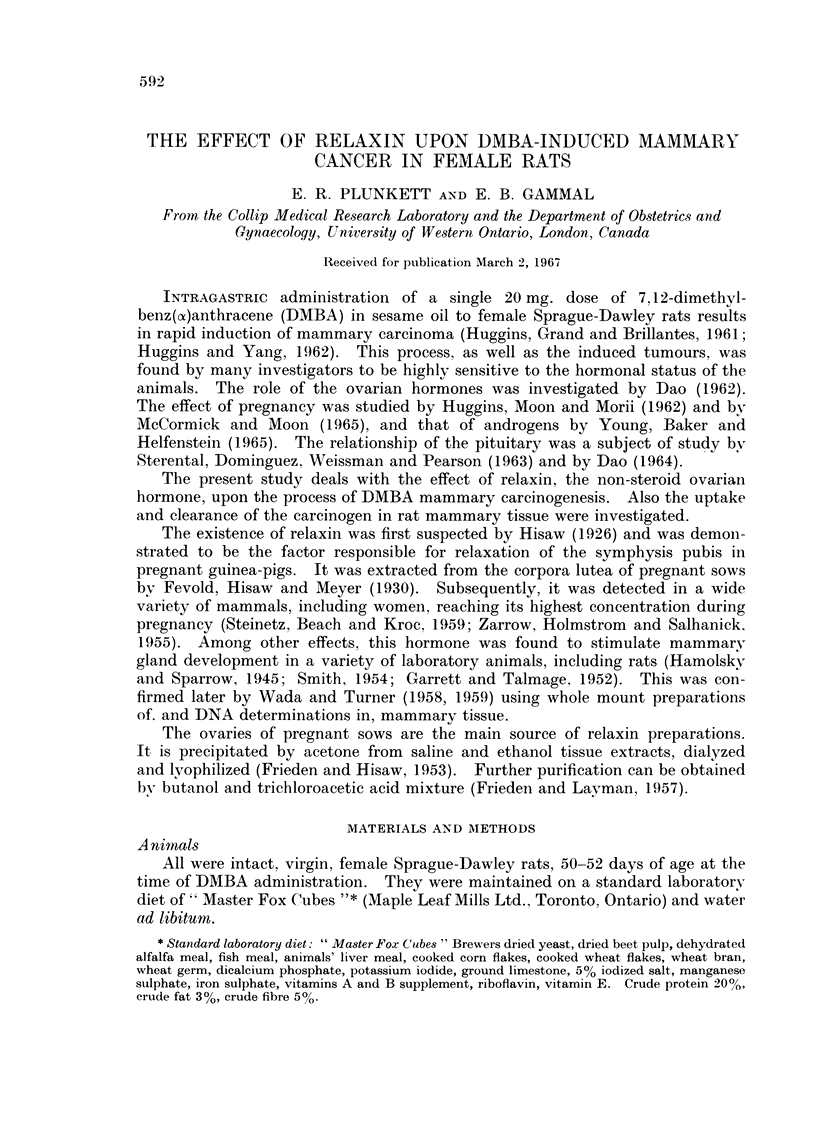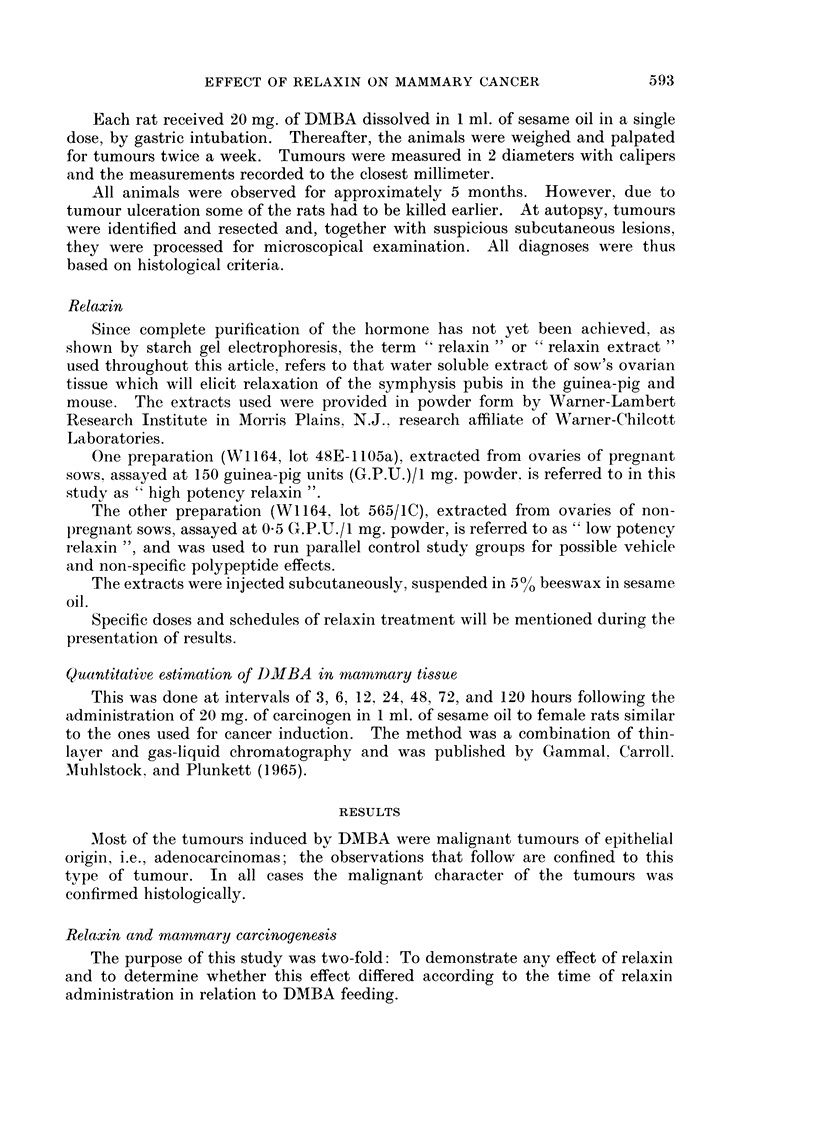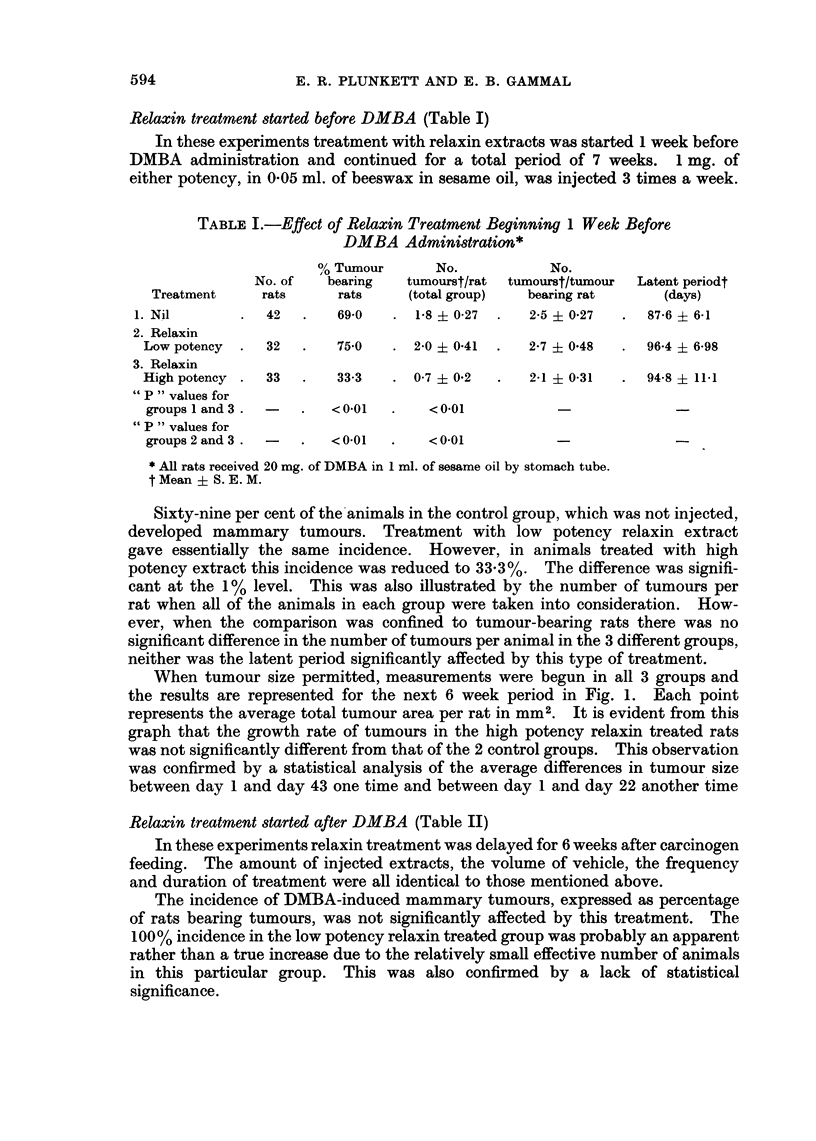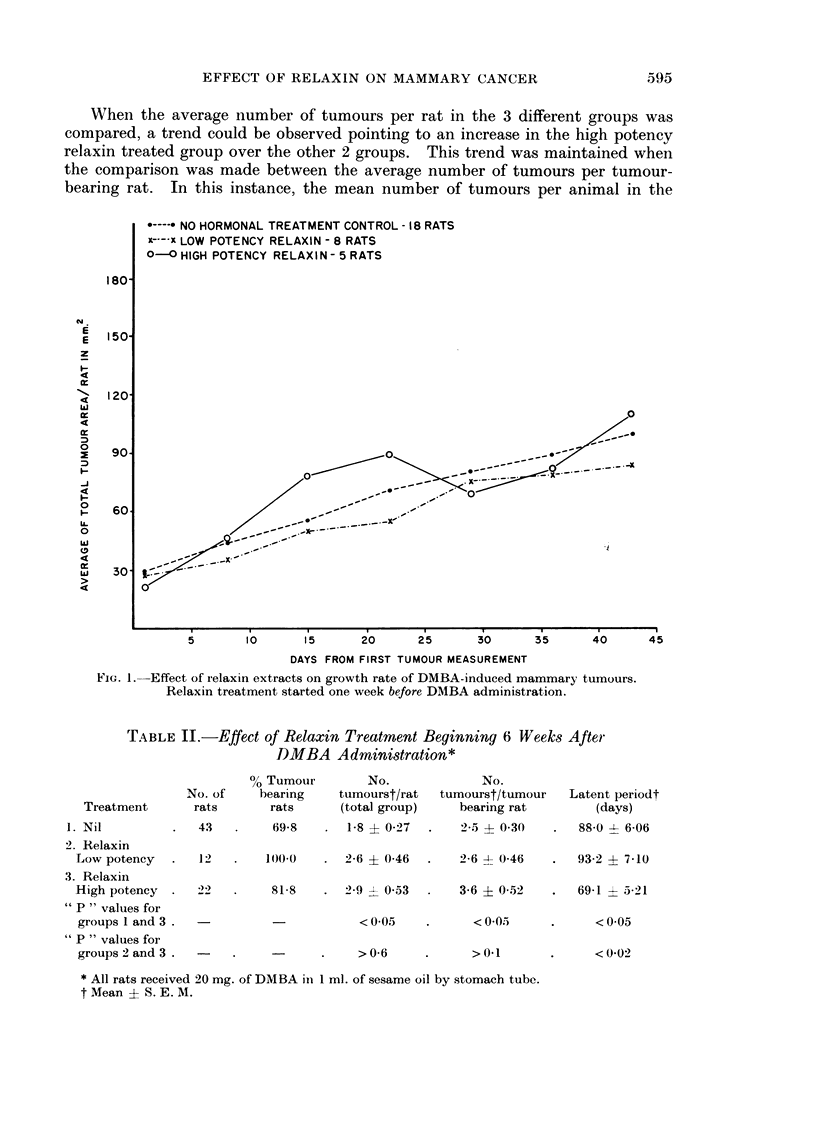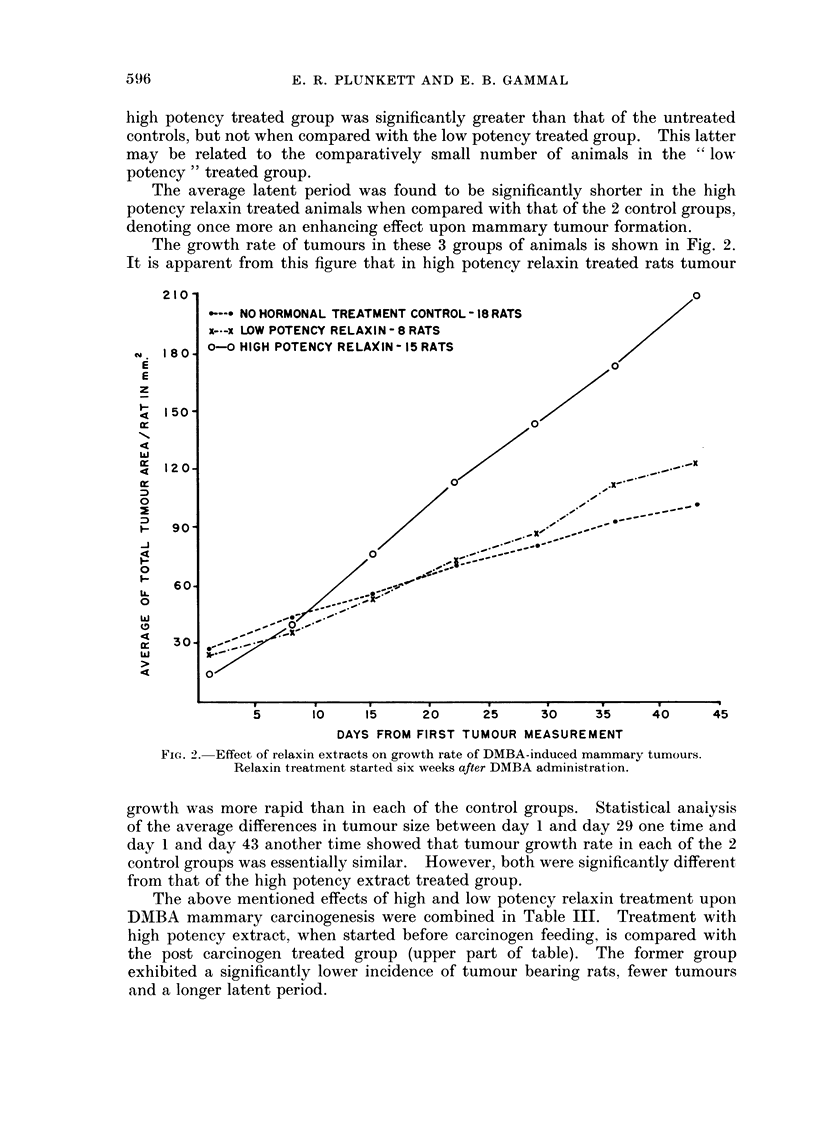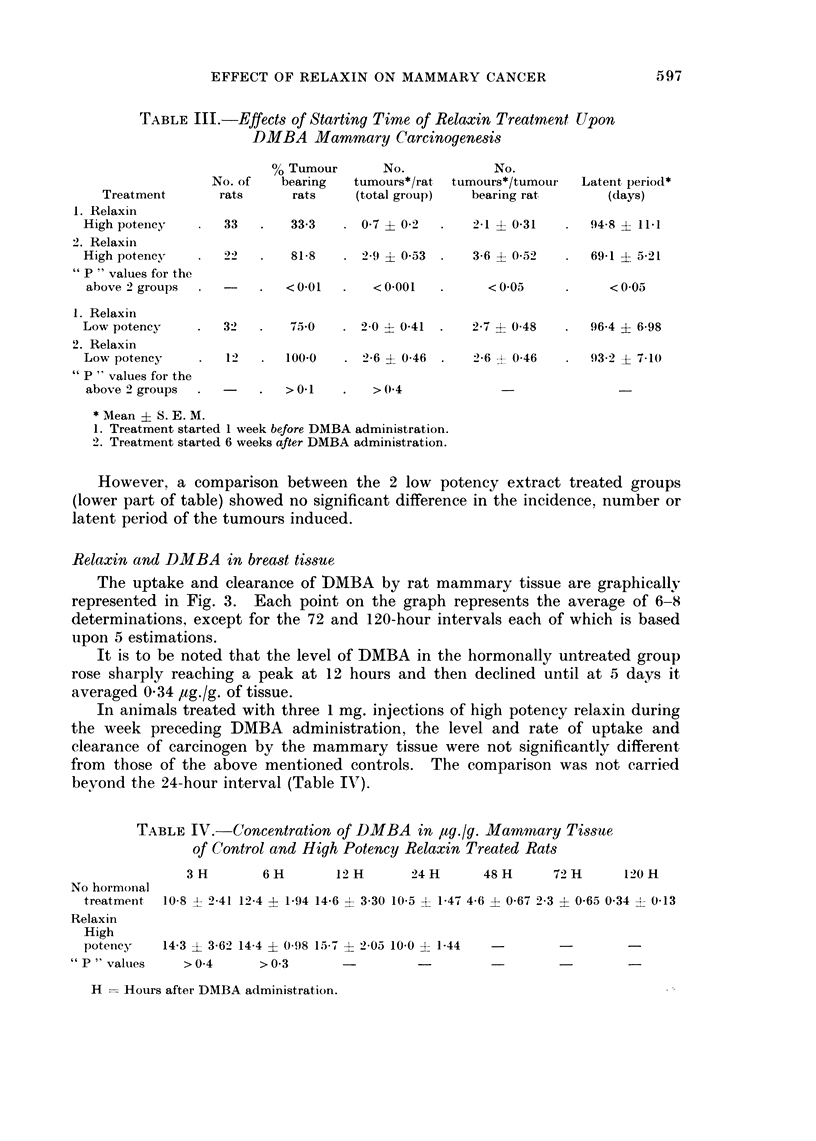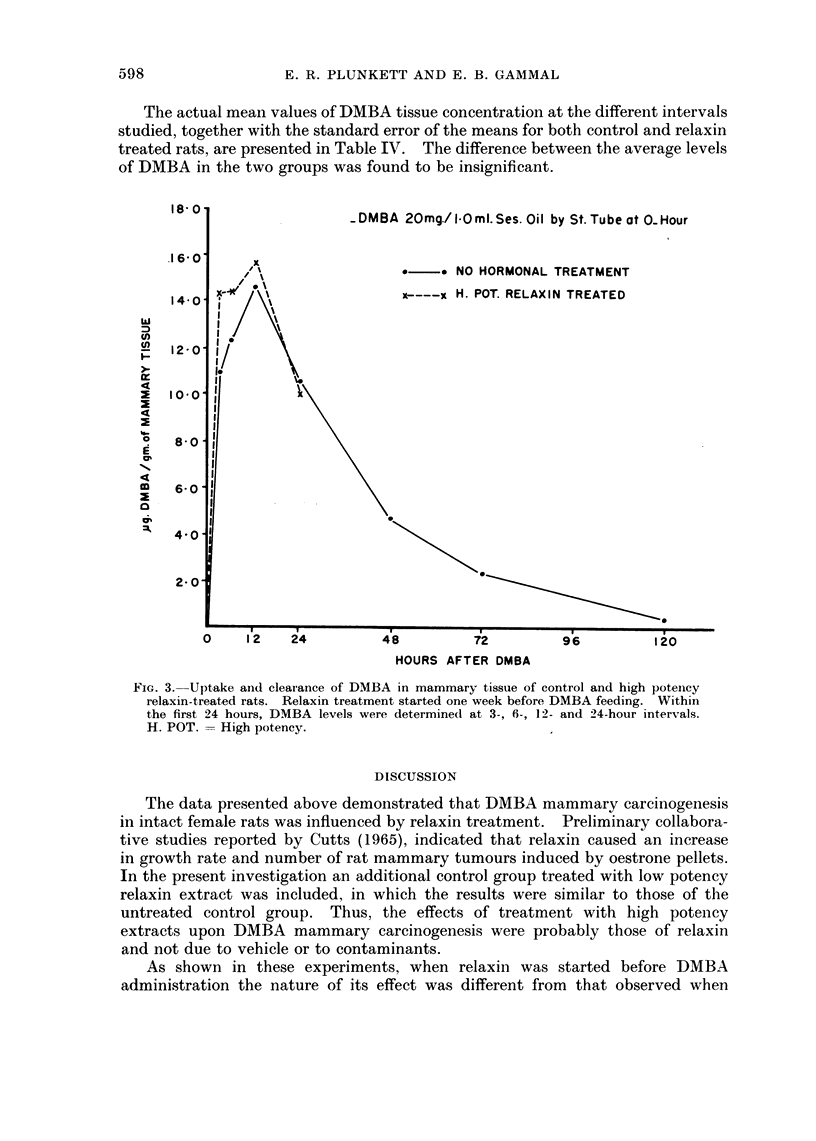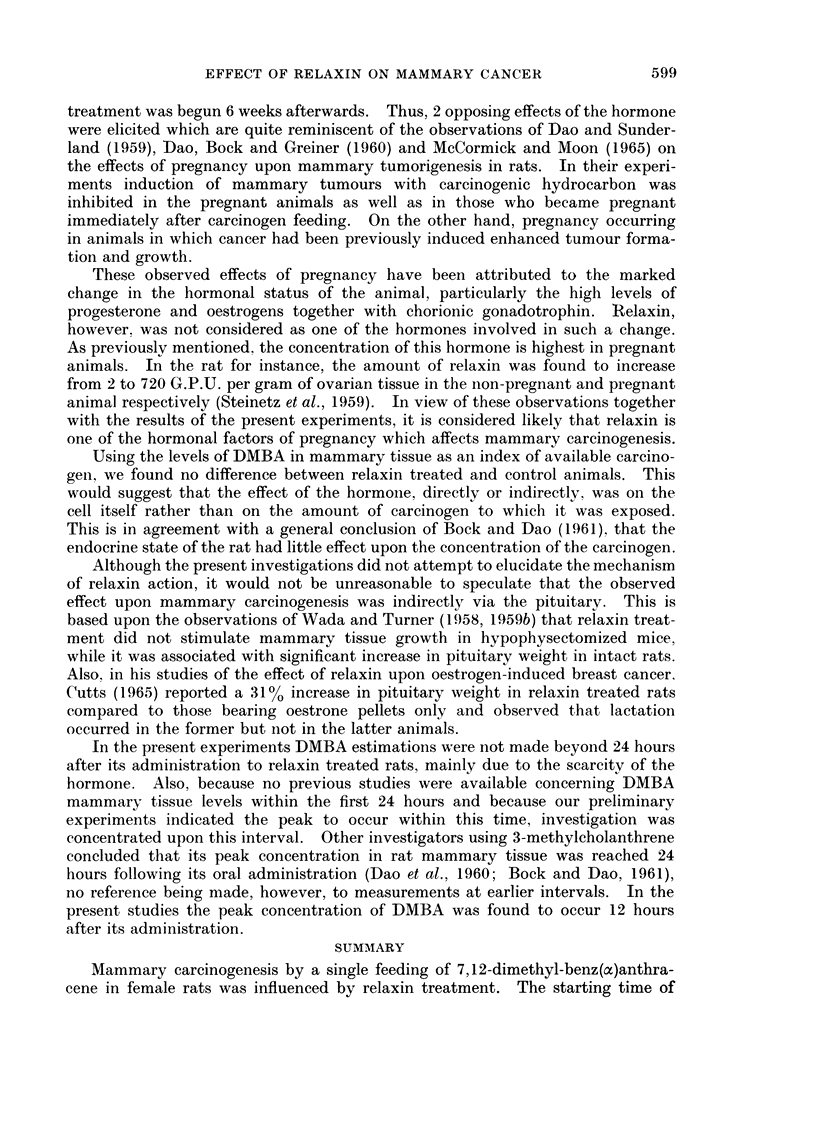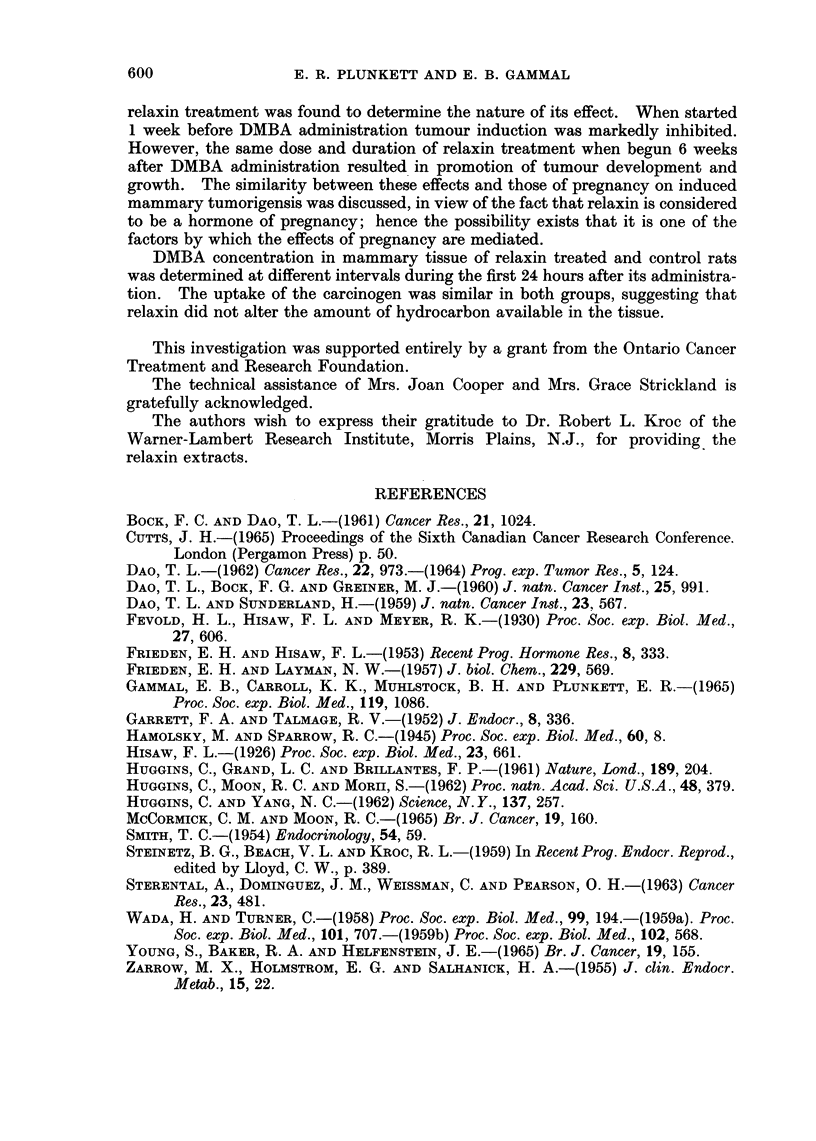# The effect of relaxin upon DMBA-induced mammary cancer in female rats.

**DOI:** 10.1038/bjc.1967.69

**Published:** 1967-09

**Authors:** E. R. Plunkett, E. B. Gammal


### THE EFFECT OF RELAXIN UPON DMBA-INDUCED MAMMARY CANCER IN FEMALE RATS

E. R. PLUNKETT AND E. B. GAMMAL

*From the Collip Medical Research Laboratory and the Department of Obstetrics and Gynaecology, University of Western Ontario, London, Canada*

Received for publication March 2, 1967

INTRAGASTRIC administration of a single 20 mg. dose of 7,12-dimethylbenz(α)anthracene (DMBA) in sesame oil to female Sprague-Dawley rats results in rapid induction of mammary carcinoma (Huggins, Grand and Brillantes, 1961; Huggins and Yang, 1962). This process, as well as the induced tumours, was found by many investigators to be highly sensitive to the hormonal status of the animals. The role of the ovarian hormones was investigated by Dao (1962). The effect of pregnancy was studied by Huggins, Moon and Morii (1962) and by McCormick and Moon (1965), and that of androgens by Young, Baker and Helfenstein (1965). The relationship of the pituitary was a subject of study by Sterental, Dominguez, Weissman and Pearson (1963) and by Dao (1964).

The present study deals with the effect of relaxin, the non-steroid ovarian hormone, upon the process of DMBA mammary carcinogenesis. Also the uptake and clearance of the carcinogen in rat mammary tissue were investigated.

The existence of relaxin was first suspected by Hisaw (1926) and was demonstrated to be the factor responsible for relaxation of the symphysis pubis in pregnant guinea-pigs. It was extracted from the corpora lutea of pregnant sows by Fevold, Hisaw and Meyer (1930). Subsequently, it was detected in a wide variety of mammals, including women, reaching its highest concentration during pregnancy (Steinetz, Beach and Kroc, 1959; Zarrow, Holmstrom and Salhanick, 1955). Among other effects, this hormone was found to stimulate mammary gland development in a variety of laboratory animals, including rats (Hamolsky and Sparrow, 1945; Smith, 1954; Garrett and Talmage, 1952). This was confirmed later by Wada and Turner (1958, 1959) using whole mount preparations of, and DNA determinations in, mammary tissue.

The ovaries of pregnant sows are the main source of relaxin preparations. It is precipitated by acetone from saline and ethanol tissue extracts, dialyzed and lyophilized (Frieden and Hisaw, 1953). Further purification can be obtained by butanol and trichloroacetic acid mixture (Frieden and Layman, 1957).

### MATERIALS AND METHODS

### *Animals*

All were intact, virgin, female Sprague-Dawley rats, 50–52 days of age at the time of DMBA administration. They were maintained on a standard laboratory diet of " Master Fox Cubes "* (Maple Leaf Mills Ltd., Toronto, Ontario) and water *ad libitum.*

* *Standard laboratory diet:* " *Master Fox Cubes* " Brewers dried yeast, dried beet pulp, dehydrated alfalfa meal, fish meal, animals' liver meal, cooked corn flakes, cooked wheat flakes, wheat bran, wheat germ, dicalcium phosphate, potassium iodide, ground limestone, 5% iodized salt, manganese sulphate, iron sulphate, vitamins A and B supplement, riboflavin, vitamin E. Crude protein 20%, crude fat 3%, crude fibre 5%.

Each rat received 20 mg. of DMBA dissolved in 1 ml. of sesame oil in a single dose, by gastric intubation. Thereafter, the animals were weighed and palpated for tumours twice a week. Tumours were measured in 2 diameters with calipers and the measurements recorded to the closest millimeter.

All animals were observed for approximately 5 months. However, due to tumour ulceration some of the rats had to be killed earlier. At autopsy, tumours were identified and resected and, together with suspicious subcutaneous lesions, they were processed for microscopical examination. All diagnoses were thus based on histological criteria.

*Relaxin*

Since complete purification of the hormone has not yet been achieved, as shown by starch gel electrophoresis, the term " relaxin " or " relaxin extract " used throughout this article, refers to that water soluble extract of sow's ovarian tissue which will elicit relaxation of the symphysis pubis in the guinea-pig and mouse. The extracts used were provided in powder form by Warner-Lambert Research Institute in Morris Plains, N.J., research affiliate of Warner-Chilcott Laboratories.

One preparation (W1164, lot 48E-1105a), extracted from ovaries of pregnant sows, assayed at 150 guinea-pig units (G.P.U.)/1 mg. powder, is referred to in this study as " high potency relaxin ".

The other preparation (W1164, lot 565/1C), extracted from ovaries of non-pregnant sows, assayed at 0·5 G.P.U./1 mg. powder, is referred to as " low potency relaxin ", and was used to run parallel control study groups for possible vehicle and non-specific polypeptide effects.

The extracts were injected subcutaneously, suspended in 5% beeswax in sesame oil.

Specific doses and schedules of relaxin treatment will be mentioned during the presentation of results.

*Quantitative estimation of DMBA in mammary tissue*

This was done at intervals of 3, 6, 12, 24, 48, 72, and 120 hours following the administration of 20 mg. of carcinogen in 1 ml. of sesame oil to female rats similar to the ones used for cancer induction. The method was a combination of thin-layer and gas-liquid chromatography and was published by Gammal, Carroll, Muhlstock, and Plunkett (1965).

### RESULTS

Most of the tumours induced by DMBA were malignant tumours of epithelial origin, i.e., adenocarcinomas; the observations that follow are confined to this type of tumour. In all cases the malignant character of the tumours was confirmed histologically.

*Relaxin and mammary carcinogenesis*

The purpose of this study was two-fold: To demonstrate any effect of relaxin and to determine whether this effect differed according to the time of relaxin administration in relation to DMBA feeding.

*Relaxin treatment started before DMBA* (Table I)

In these experiments treatment with relaxin extracts was started 1 week before DMBA administration and continued for a total period of 7 weeks. 1 mg. of either potency, in 0·05 ml. of beeswax in sesame oil, was injected 3 times a week.

TABLE I.—*Effect of Relaxin Treatment Beginning 1 Week Before DMBA Administration**

| Treatment | No. of rats | % Tumour bearing rats | No. tumours†/rat (total group) | No. tumours†/tumour bearing rat | Latent period† (days) |
|---|---|---|---|---|---|
| 1. Nil | 42 | 69·0 | 1·8 ± 0·27 | 2·5 ± 0·27 | 87·6 ± 6·1 |
| 2. Relaxin Low potency | 32 | 75·0 | 2·0 ± 0·41 | 2·7 ± 0·48 | 96·4 ± 6·98 |
| 3. Relaxin High potency | 33 | 33·3 | 0·7 ± 0·2 | 2·1 ± 0·31 | 94·8 ± 11·1 |
| " P " values for groups 1 and 3 | — | < 0·01 | < 0·01 | — | — |
| " P " values for groups 2 and 3 | — | < 0·01 | < 0·01 | — | — |

* All rats received 20 mg. of DMBA in 1 ml. of sesame oil by stomach tube.
† Mean ± S. E. M.

Sixty-nine per cent of the animals in the control group, which was not injected, developed mammary tumours. Treatment with low potency relaxin extract gave essentially the same incidence. However, in animals treated with high potency extract this incidence was reduced to 33·3%. The difference was significant at the 1% level. This was also illustrated by the number of tumours per rat when all of the animals in each group were taken into consideration. However, when the comparison was confined to tumour-bearing rats there was no significant difference in the number of tumours per animal in the 3 different groups, neither was the latent period significantly affected by this type of treatment.

When tumour size permitted, measurements were begun in all 3 groups and the results are represented for the next 6 week period in Fig. 1. Each point represents the average total tumour area per rat in mm$^2$. It is evident from this graph that the growth rate of tumours in the high potency relaxin treated rats was not significantly different from that of the 2 control groups. This observation was confirmed by a statistical analysis of the average differences in tumour size between day 1 and day 43 one time and between day 1 and day 22 another time

*Relaxin treatment started after DMBA* (Table II)

In these experiments relaxin treatment was delayed for 6 weeks after carcinogen feeding. The amount of injected extracts, the volume of vehicle, the frequency and duration of treatment were all identical to those mentioned above.

The incidence of DMBA-induced mammary tumours, expressed as percentage of rats bearing tumours, was not significantly affected by this treatment. The 100% incidence in the low potency relaxin treated group was probably an apparent rather than a true increase due to the relatively small effective number of animals in this particular group. This was also confirmed by a lack of statistical significance.

When the average number of tumours per rat in the 3 different groups was compared, a trend could be observed pointing to an increase in the high potency relaxin treated group over the other 2 groups. This trend was maintained when the comparison was made between the average number of tumours per tumour-bearing rat. In this instance, the mean number of tumours per animal in the

FIG. 1.—Effect of relaxin extracts on growth rate of DMBA-induced mammary tumours. Relaxin treatment started one week *before* DMBA administration.

TABLE II.—*Effect of Relaxin Treatment Beginning 6 Weeks After DMBA Administration**

| Treatment | No. of rats | % Tumour bearing rats | No. tumours†/rat (total group) | No. tumours†/tumour bearing rat | Latent period† (days) |
|---|---|---|---|---|---|
| 1. Nil | 43 | 69·8 | 1·8 ± 0·27 | 2·5 ± 0·30 | 88·0 ± 6·06 |
| 2. Relaxin Low potency | 12 | 100·0 | 2·6 ± 0·46 | 2·6 ± 0·46 | 93·2 ± 7·10 |
| 3. Relaxin High potency | 22 | 81·8 | 2·9 ± 0·53 | 3·6 ± 0·52 | 69·1 ± 5·21 |
| " P " values for groups 1 and 3 | — | — | < 0·05 | < 0·05 | < 0·05 |
| " P " values for groups 2 and 3 | — | — | > 0·6 | > 0·1 | < 0·02 |

* All rats received 20 mg. of DMBA in 1 ml. of sesame oil by stomach tube.
† Mean ± S. E. M.

high potency treated group was significantly greater than that of the untreated controls, but not when compared with the low potency treated group. This latter may be related to the comparatively small number of animals in the " low potency " treated group.

The average latent period was found to be significantly shorter in the high potency relaxin treated animals when compared with that of the 2 control groups, denoting once more an enhancing effect upon mammary tumour formation.

The growth rate of tumours in these 3 groups of animals is shown in Fig. 2. It is apparent from this figure that in high potency relaxin treated rats tumour

FIG. 2.—Effect of relaxin extracts on growth rate of DMBA-induced mammary tumours. Relaxin treatment started six weeks *after* DMBA administration.

growth was more rapid than in each of the control groups. Statistical analysis of the average differences in tumour size between day 1 and day 29 one time and day 1 and day 43 another time showed that tumour growth rate in each of the 2 control groups was essentially similar. However, both were significantly different from that of the high potency extract treated group.

The above mentioned effects of high and low potency relaxin treatment upon DMBA mammary carcinogenesis were combined in Table III. Treatment with high potency extract, when started before carcinogen feeding, is compared with the post carcinogen treated group (upper part of table). The former group exhibited a significantly lower incidence of tumour bearing rats, fewer tumours and a longer latent period.

TABLE III.—*Effects of Starting Time of Relaxin Treatment Upon DMBA Mammary Carcinogenesis*

| Treatment | No. of rats | % Tumour bearing rats | No. tumours*/rat (total group) | No. tumours*/tumour bearing rat | Latent period* (days) |
|---|---|---|---|---|---|
| 1. Relaxin High potency | 33 | 33·3 | 0·7 ± 0·2 | 2·1 ± 0·31 | 94·8 ± 11·1 |
| 2. Relaxin High potency | 22 | 81·8 | 2·9 ± 0·53 | 3·6 ± 0·52 | 69·1 ± 5·21 |
| " P " values for the above 2 groups | — | < 0·01 | < 0·001 | < 0·05 | < 0·05 |
| 1. Relaxin Low potency | 32 | 75·0 | 2·0 ± 0·41 | 2·7 ± 0·48 | 96·4 ± 6·98 |
| 2. Relaxin Low potency | 12 | 100·0 | 2·6 ± 0·46 | 2·6 ± 0·46 | 93·2 ± 7·10 |
| " P " values for the above 2 groups | — | > 0·1 | > 0·4 | — | — |

* Mean ± S. E. M.
1. Treatment started 1 week *before* DMBA administration.
2. Treatment started 6 weeks *after* DMBA administration.

However, a comparison between the 2 low potency extract treated groups (lower part of table) showed no significant difference in the incidence, number or latent period of the tumours induced.

*Relaxin and DMBA in breast tissue*

The uptake and clearance of DMBA by rat mammary tissue are graphically represented in Fig. 3. Each point on the graph represents the average of 6–8 determinations, except for the 72 and 120-hour intervals each of which is based upon 5 estimations.

It is to be noted that the level of DMBA in the hormonally untreated group rose sharply reaching a peak at 12 hours and then declined until at 5 days it averaged 0·34 $\mu$g./g. of tissue.

In animals treated with three 1 mg. injections of high potency relaxin during the week preceding DMBA administration, the level and rate of uptake and clearance of carcinogen by the mammary tissue were not significantly different from those of the above mentioned controls. The comparison was not carried beyond the 24-hour interval (Table IV).

TABLE IV.—*Concentration of DMBA in $\mu$g./g. Mammary Tissue of Control and High Potency Relaxin Treated Rats*

| | 3 H | 6 H | 12 H | 24 H | 48 H | 72 H | 120 H |
|---|---|---|---|---|---|---|---|
| No hormonal treatment | 10·8 ± 2·41 | 12·4 ± 1·94 | 14·6 ± 3·30 | 10·5 ± 1·47 | 4·6 ± 0·67 | 2·3 ± 0·65 | 0·34 ± 0·13 |
| Relaxin High potency | 14·3 ± 3·62 | 14·4 ± 0·98 | 15·7 ± 2·05 | 10·0 ± 1·44 | — | — | — |
| " P " values | > 0·4 | > 0·3 | — | — | — | — | — |

H = Hours after DMBA administration.

The actual mean values of DMBA tissue concentration at the different intervals studied, together with the standard error of the means for both control and relaxin treated rats, are presented in Table IV. The difference between the average levels of DMBA in the two groups was found to be insignificant.

FIG. 3.—Uptake and clearance of DMBA in mammary tissue of control and high potency relaxin-treated rats. Relaxin treatment started one week before DMBA feeding. Within the first 24 hours, DMBA levels were determined at 3-, 6-, 12- and 24-hour intervals. H. POT. = High potency.

### DISCUSSION

The data presented above demonstrated that DMBA mammary carcinogenesis in intact female rats was influenced by relaxin treatment. Preliminary collaborative studies reported by Cutts (1965), indicated that relaxin caused an increase in growth rate and number of rat mammary tumours induced by oestrone pellets. In the present investigation an additional control group treated with low potency relaxin extract was included, in which the results were similar to those of the untreated control group. Thus, the effects of treatment with high potency extracts upon DMBA mammary carcinogenesis were probably those of relaxin and not due to vehicle or to contaminants.

As shown in these experiments, when relaxin was started before DMBA administration the nature of its effect was different from that observed when treatment was begun 6 weeks afterwards. Thus, 2 opposing effects of the hormone were elicited which are quite reminiscent of the observations of Dao and Sunderland (1959), Dao, Bock and Greiner (1960) and McCormick and Moon (1965) on the effects of pregnancy upon mammary tumorigenesis in rats. In their experiments induction of mammary tumours with carcinogenic hydrocarbon was inhibited in the pregnant animals as well as in those who became pregnant immediately after carcinogen feeding. On the other hand, pregnancy occurring in animals in which cancer had been previously induced enhanced tumour formation and growth.

These observed effects of pregnancy have been attributed to the marked change in the hormonal status of the animal, particularly the high levels of progesterone and oestrogens together with chorionic gonadotrophin. Relaxin, however, was not considered as one of the hormones involved in such a change. As previously mentioned, the concentration of this hormone is highest in pregnant animals. In the rat for instance, the amount of relaxin was found to increase from 2 to 720 G.P.U. per gram of ovarian tissue in the non-pregnant and pregnant animal respectively (Steinetz *et al.*, 1959). In view of these observations together with the results of the present experiments, it is considered likely that relaxin is one of the hormonal factors of pregnancy which affects mammary carcinogenesis.

Using the levels of DMBA in mammary tissue as an index of available carcinogen, we found no difference between relaxin treated and control animals. This would suggest that the effect of the hormone, directly or indirectly, was on the cell itself rather than on the amount of carcinogen to which it was exposed. This is in agreement with a general conclusion of Bock and Dao (1961), that the endocrine state of the rat had little effect upon the concentration of the carcinogen.

Although the present investigations did not attempt to elucidate the mechanism of relaxin action, it would not be unreasonable to speculate that the observed effect upon mammary carcinogenesis was indirectly via the pituitary. This is based upon the observations of Wada and Turner (1958, 1959*b*) that relaxin treatment did not stimulate mammary tissue growth in hypophysectomized mice, while it was associated with significant increase in pituitary weight in intact rats. Also, in his studies of the effect of relaxin upon oestrogen-induced breast cancer, Cutts (1965) reported a 31% increase in pituitary weight in relaxin treated rats compared to those bearing oestrone pellets only and observed that lactation occurred in the former but not in the latter animals.

In the present experiments DMBA estimations were not made beyond 24 hours after its administration to relaxin treated rats, mainly due to the scarcity of the hormone. Also, because no previous studies were available concerning DMBA mammary tissue levels within the first 24 hours and because our preliminary experiments indicated the peak to occur within this time, investigation was concentrated upon this interval. Other investigators using 3-methylcholanthrene concluded that its peak concentration in rat mammary tissue was reached 24 hours following its oral administration (Dao *et al.*, 1960; Bock and Dao, 1961), no reference being made, however, to measurements at earlier intervals. In the present studies the peak concentration of DMBA was found to occur 12 hours after its administration.

### SUMMARY

Mammary carcinogenesis by a single feeding of 7,12-dimethyl-benz($\alpha$)anthracene in female rats was influenced by relaxin treatment. The starting time of relaxin treatment was found to determine the nature of its effect. When started 1 week before DMBA administration tumour induction was markedly inhibited. However, the same dose and duration of relaxin treatment when begun 6 weeks after DMBA administration resulted in promotion of tumour development and growth. The similarity between these effects and those of pregnancy on induced mammary tumorigensis was discussed, in view of the fact that relaxin is considered to be a hormone of pregnancy; hence the possibility exists that it is one of the factors by which the effects of pregnancy are mediated.

DMBA concentration in mammary tissue of relaxin treated and control rats was determined at different intervals during the first 24 hours after its administration. The uptake of the carcinogen was similar in both groups, suggesting that relaxin did not alter the amount of hydrocarbon available in the tissue.

This investigation was supported entirely by a grant from the Ontario Cancer Treatment and Research Foundation.

The technical assistance of Mrs. Joan Cooper and Mrs. Grace Strickland is gratefully acknowledged.

The authors wish to express their gratitude to Dr. Robert L. Kroc of the Warner-Lambert Research Institute, Morris Plains, N.J., for providing the relaxin extracts.

### REFERENCES

Bock, F. C. and Dao, T. L.—(1961) *Cancer Res.*, **21**, 1024.
Cutts, J. H.—(1965) Proceedings of the Sixth Canadian Cancer Research Conference. London (Pergamon Press) p. 50.
Dao, T. L.—(1962) *Cancer Res.*, **22**, 973.—(1964) *Prog. exp. Tumor Res.*, **5**, 124.
Dao, T. L., Bock, F. G. and Greiner, M. J.—(1960) *J. natn. Cancer Inst.*, **25**, 991.
Dao, T. L. and Sunderland, H.—(1959) *J. natn. Cancer Inst.*, **23**, 567.
Fevold, H. L., Hisaw, F. L. and Meyer, R. K.—(1930) *Proc. Soc. exp. Biol. Med.*, **27**, 606.
Frieden, E. H. and Hisaw, F. L.—(1953) *Recent Prog. Hormone Res.*, **8**, 333.
Frieden, E. H. and Layman, N. W.—(1957) *J. biol. Chem.*, **229**, 569.
Gammal, E. B., Carroll, K. K., Muhlstock, B. H. and Plunkett, E. R.—(1965) *Proc. Soc. exp. Biol. Med.*, **119**, 1086.
Garrett, F. A. and Talmage, R. V.—(1952) *J. Endocr.*, **8**, 336.
Hamolsky, M. and Sparrow, R. C.—(1945) *Proc. Soc. exp. Biol. Med.*, **60**, 8.
Hisaw, F. L.—(1926) *Proc. Soc. exp. Biol. Med.*, **23**, 661.
Huggins, C., Grand, L. C. and Brillantes, F. P.—(1961) *Nature, Lond.*, **189**, 204.
Huggins, C., Moon, R. C. and Morii, S.—(1962) *Proc. natn. Acad. Sci. U.S.A.*, **48**, 379.
Huggins, C. and Yang, N. C.—(1962) *Science, N.Y.*, **137**, 257.
McCormick, C. M. and Moon, R. C.—(1965) *Br. J. Cancer*, **19**, 160.
Smith, T. C.—(1954) *Endocrinology*, **54**, 59.
Steinetz, B. G., Beach, V. L. and Kroc, R. L.—(1959) In *Recent Prog. Endocr. Reprod.*, edited by Lloyd, C. W., p. 389.
Sterental, A., Dominguez, J. M., Weissman, C. and Pearson, O. H.—(1963) *Cancer Res.*, **23**, 481.
Wada, H. and Turner, C.—(1958) *Proc. Soc. exp. Biol. Med.*, **99**, 194.—(1959a). *Proc. Soc. exp. Biol. Med.*, **101**, 707.—(1959b) *Proc. Soc. exp. Biol. Med.*, **102**, 568.
Young, S., Baker, R. A. and Helfenstein, J. E.—(1965) *Br. J. Cancer*, **19**, 155.
Zarrow, M. X., Holmstrom, E. G. and Salhanick, H. A.—(1955) *J. clin. Endocr. Metab.*, **15**, 22.